# Non‐Invasive Measures of DNA Methylation Capture Molecular Aging in Wild Capuchin Monkeys

**DOI:** 10.1111/mec.70468

**Published:** 2026-07-15

**Authors:** Baptiste Sadoughi, Raquel Hernández‐Rojas, Hadjira Hamou, Ronald Lopez, Megan Mah, Elizabeth Slikas, Sierra M. V. Simmons, Joseph D. Orkin, James P. Higham, Sarah F. Brosnan, Katharine M. Jack, Fernando A. Campos, Noah Snyder‐Mackler, Amanda D. Melin

**Affiliations:** ^1^ Center for Evolution and Medicine Arizona State University Tempe Arizona USA; ^2^ School of Life Sciences Arizona State University Tempe Arizona USA; ^3^ Department of Anthropology and Archaeology University of Calgary Calgary Canada; ^4^ Santa Rosa Primate Project Área de Conservación Guanacaste Liberia Costa Rica; ^5^ Department of Psychology and Language Research Center Georgia State University Atlanta Georgia USA; ^6^ Département D'anthropologie Université de Montréal Montréal Quebec Canada; ^7^ Département de Sciences Biologiques Université de Montréal Montréal Quebec Canada; ^8^ Department of Anthropology New York University New York New York USA; ^9^ Department of Anthropology Tulane University New Orleans Louisiana USA; ^10^ Department of Anthropology University of Texas at San Antonio San Antonio Texas USA; ^11^ School for Human Evolution and Social Change Arizona State University Tempe Arizona USA; ^12^ Department of Medical Genetics University of Calgary Calgary Alberta Canada; ^13^ Alberta Children's Hospital Research Institute University of Calgary Calgary Alberta Canada

**Keywords:** age estimation, aging hallmarks, epigenetic clock, non‐invasive epigenomics, primate

## Abstract

Elucidating the socio‐ecological factors that shape patterns of aging in long‐lived vertebrates is of broad interest to evolutionary biologists, geroscientists, and ecologists. However, research on aging in wild populations still faces challenges due to the difficulty of measuring cellular hallmarks of aging non‐invasively and the difficulty of accurately estimating age in long‐lived species. Here, we demonstrate that cellular DNA methylation (DNAm) profiles from faecal samples provide an accurate and reliable molecular clock for the estimation of age in a population of wild capuchins, a non‐human primate species with exceptional longevity. Analysis of blood, faeces, and urine samples from a closely related species shows that DNAm differentiates between species and different types of biological samples, validating their biological specificity. We further find age‐associated differences in DNAm relevant to development, membrane excitability, and control of the cell cycle. By generating new data on molecular aging in a long‐lived, socially complex primate and demonstrating that DNAm can be studied non‐invasively in wild animals, our research opens up new avenues for studying the socioecological determinants of aging and increases the potential for cross‐population and cross‐species comparisons.

## 1 Introduction

Aging is a complex and multifaceted process that impacts health, reproduction and survival—key contributors to fitness. Strikingly, individuals of the same chronological age often differ markedly in the onset, manifestation, and pace of age‐related decline (Avila‐Rieger et al. 2022; Bouwhuis et al. 2010; Fiorito et al. 2017; Jones et al. 2014; Kivimäki et al. 2025). Understanding this heterogeneity is a central goal in aging research and has motivated the development of molecular hallmarks and biomarkers that track age‐associated changes across individuals. However, studying variation in aging trajectories requires accurate knowledge or estimation of chronological age, which is often unavailable in natural populations. Long‐term field studies have advanced biogerontology (Nussey et al. 2013), but ages remain frequently unknown for some individuals (e.g., immigrants into study populations, individuals who were alive at the onset of studies). Consequently, reliable biomarkers for estimating chronological age are immensely helpful for advancing aging research.

Among candidate biomarkers, DNA methylation (DNAm) is considered one of the most robust molecular indicators of age‐related variation across tissues and individuals. DNAm refers to the addition of methyl groups to cytosine bases, typically within CpG dinucleotides in mammals. As an epigenetic mechanism, DNAm plays a central role in regulating gene expression (Petersen et al. 2025) and is essential for development and tissue differentiation (Greenberg and Bourc'his 2019; Loyfer et al. 2023). It is also implicated in physiological decline and age‐related disease (Hernando‐Herraez et al. 2019; Horvath and Raj 2018; Kabacik et al. 2022; Yang et al. 2023). Importantly, DNAm exhibits predictable changes with age, making it a powerful tool for both estimating chronological age and investigating the molecular correlates of aging.

In humans, DNAm data are widely used to construct epigenetic ‘clocks’, predictive models trained on methylation profiles to estimate chronological age (Hannum et al. 2013; Horvath 2013), mortality risk (Levine et al. 2018; Lu et al. 2019), or the pace of aging (Belsky et al. 2022). First‐generation clocks optimized to predict chronological age are particularly relevant for estimating unknown ages (Horvath and Raj 2018; Johnson and Shokhirev 2025). Complementing these approaches, epigenome‐wide association studies (EWAS) identify loci where methylation changes systematically with age or disease, providing insights into age‐related shifts in gene regulation (Hernando‐Herraez et al. 2019; Marttila et al. 2015; Reynolds et al. 2014) and enabling the detection of tissue‐specific pathologies (Chen et al. 2009; Li et al. 2023; Schlosser et al. 2021; Zemmour et al. 2018). Together, DNAm‐based clocks and EWAS highlight the dual predictive and exploratory utility of DNAm in aging research.

In contrast, research on DNAm and aging in non‐human organisms remains comparatively limited, though it is rapidly expanding. Recent studies have developed DNAm clocks for individual species and across taxa (Le Clercq et al. 2023; McCoy et al. 2024; Robeck et al. 2021, 2023; Thompson et al. 2017; Weber et al. 2024; Wilkinson et al. 2021), including panmammalian clocks capable of predicting age with high accuracy relative to lifespan (e.g., to within 3.3% error relative to the lifespan (Lu et al. 2023)). These approaches enable accurate estimation of chronological age where it is otherwise unknown—a common challenge in wildlife studies (Le Clercq et al. 2023; Newediuk et al. 2025). In addition, they enable identification of conserved genomic regions associated with aging and characterization of regulatory features (e.g., chromatin states, transcription factor binding sites, transposable elements) exhibiting variation in methylation levels (Kerepesi et al. 2022; Lu et al. 2023; Wilkinson et al. 2021). Taking epigenetic research out of the lab and into the field is critical for understanding how environmental variation shapes aging. DNAm patterns in natural populations provide insight into phenotypic diversity and plasticity in response to environmental exposures (Anderson et al. 2024; Goldman and Sterner 2023; Meuthen et al. 2025). Such field‐based approaches broaden the taxonomic scope of aging research and place human aging within an eco‐evolutionary framework (Newediuk et al. 2025; Nussey 2025; Roach and Carey 2014). However, DNAm measurement has heretofore required invasive sampling (e.g., blood or tissue), limiting its application in wild and non‐model species. As a result, we are missing critical opportunities to study environmental and ecological influences on aging across the tree of life.

Non‐human primates are long‐lived and ecologically and socially diverse, with a growing pool of genomic resources that make them valuable for comparative research across disciplines from anthropology to biomedicine (Fiziev et al. 2023; Rocha et al. 2024). Their close evolutionary history with humans and broadly similar aging (Bronikowski et al. 2011; Colchero et al. 2021) make them a highly translational system for studying aging heterogeneity (Campos et al. 2024; Emery Thompson et al. 2020; Fernandes et al. 2025; Newman et al. 2023; Tung et al. 2023; Wilson et al. 2020). Long‐running studies of non‐human primates, where molecular, social, and ecological environments are deeply documented, are providing key insights into the ecological and social determinants of health and aging (Campos et al. 2024; Emery Thompson et al. 2020; Newman et al. 2023; Sadoughi et al. 2022; Tung et al. 2023; Wilson et al. 2020). In particular, naturalistic and wild systems reveal how environmental variation, both long‐lasting such as drought and abrupt such as hurricanes, alter the relationships between socio‐ecological factors, age, and survival (Anderson et al. 2024; Testard et al. 2024). However, realizing the full potential of comparative approaches requires broader representation of species in their natural ecological contexts (Nussey 2025). Since many wild populations cannot be sampled regularly for blood or other tissues, methods to quantify the epigenome using non‐invasive samples are desperately needed.

**FIGURE 1:**
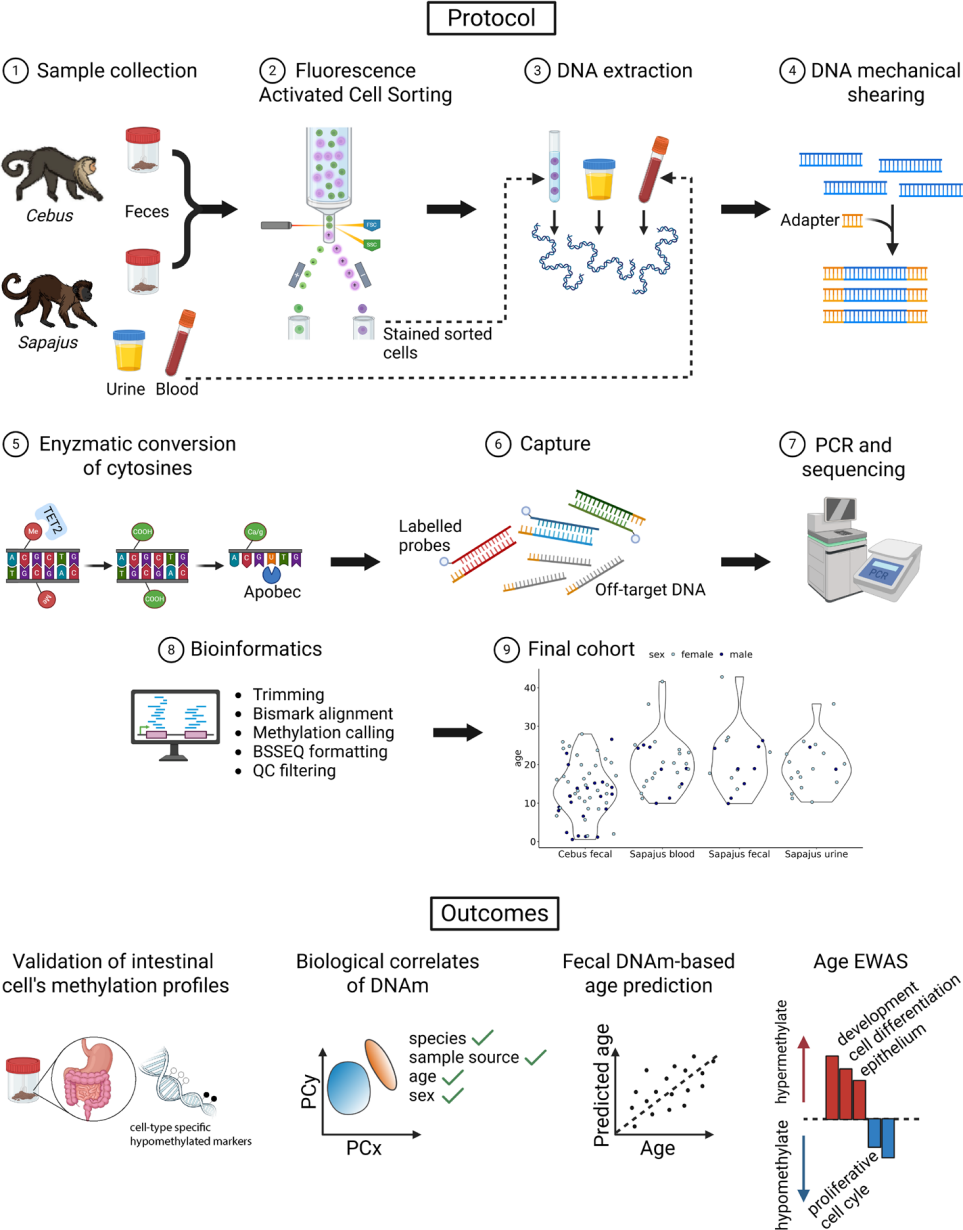
Study design and main outcomes. After collecting faecal samples from wild capuchins ( *Cebus imitator* ), and faecal, urine, and blood samples from captive capuchins ( *Sapajus apella* ), we used a flow cytometry based cell sorting protocol (Orkin et al. 2021) to enrich material recovered from faecal samples in capuchin DNA and remove contaminants. We extracted DNA from sorted faecal cells and from blood and urine samples directly, prior to enzymatic‐based methylation sequencing. Following bioinformatics and quality check filtering on libraries, the final cohort included 116 samples across three sample sources and two species. Faecal‐derived methylation profiles exhibit signatures of gut tissue specificity, species, age, and sex and can be leveraged to build accurate methylation clocks. Age‐associated differences in methylation from faecal samples hint at conserved variation at a multitude of genomic locations involving housekeeping genes, developmental processes, and immune responses. *Cebus* and *Sapajus* artwork by Jordie Hoffman; organs icons from AdobeStock; created with Biorender.com.

Here, we developed and optimized a protocol for quantifying DNAm from non‐invasively collected faecal samples in wild white‐faced capuchin monkeys ( *Cebus imitator* ), a species notable for its exceptional longevity relative to body size (up to 54 years in captivity and 37 years in the wild; 3–5 kg) (Melin et al. 2020). Our aims were to: (1) validate that faecal DNAm profiles reflect the expected tissue source (intestinal epithelium); (2) investigate variation in DNAm across different types of biological samples (blood, faecal, and urine), two closely related capuchin species, and sexes; (3) evaluate the accuracy of DNAm‐based estimation of chronological age; and (4) perform an EWAS of age‐related variation in faecal DNAm in capuchins (Figure 1).

## 2 Materials and Methods

### 2.1 Study Populations and Sampling

This study adheres to the guidelines of the American Society of Primatologists and the International Primatological Society. Permit details for the study and sample export are provided in the Ethics Statement at the end of the manuscript. We collected faecal samples from 53 habituated, wild white‐faced capuchins ( *Cebus imitator* ) in Sector Santa Rosa, Área de Conservación Guanacaste, Costa Rica. Capuchins in this population have been studied nearly continuously since the Santa Rosa Primate Project was initiated in 1983 (reviewed in Melin et al. 2020). All individuals were individually identified through fur patterns, scars, and natural morphological variation. We sampled 20 males and 33 females. Most individuals (42/53) have been followed since birth and their date of birth is known to within 1 day—ca. 1 month. One female first seen as an infant had only a known year of birth. We considered ages to be known for these 43 individuals. Ages ranged from 6 months to 26 years old in males, and from 1 year old to 24 years old in females. Other individuals (males *N* = 7, females *N* = 3) were tracked from the time they were first observed as subadults or adults (for males, typically when they immigrated into the study population; Table S1). An experienced researcher estimated the ages of these 10 individuals based on body size and fullness, limb proportions, facial width and length, prominence of brow ridge, length of eyebrows and nipples (females) (Chapoy et al. 2025), and we excluded them from all analyses including age as a predictor. Approximately 1 g of faeces was collected from forest substrates immediately following defecation by trained and experienced researchers wearing nitrile gloves and a face mask, and transferred into a 5 mL conical tube containing 2.5 mL of RNAlater. The samples were stored at room temperature until they were shipped to the University of Calgary for processing.

Brown capuchins ( *Sapajus apella* ) were members of the captive capuchin monkey colony at Georgia State University. This colony was originally formed in 2006 and currently contains 8 males (ages 11–26 at the time of sample collection) and 20 females (ages 22–42 at the time of sample collection). Monkeys live in mixed‐sex social groups, excepting one bachelor pair of males, and most monkeys have lived with their group mates their entire lives. Each group, including the bachelor pair, has a dedicated indoor room and outdoor yard to which they have access except during voluntary testing and inclement weather. Monkeys are fed a species‐typical diet including monkey chow, fruits, vegetables, and treats, and monkeys have access to water *ad libitum*. Urine and faecal samples were collected from clean trays placed beneath the monkeys' testing areas during voluntary behavioural and cognitive testing for other research. Samples were placed in 5 mL of RNAlater and stored at room temperature until they were shipped to the University of Calgary for processing. Monkeys are never restricted from food, water, treats, outdoor access, or social contact to motivate participation in research; as a result, urine and faecal samples were only available from monkeys who chose to participate in testing. Whole blood samples were collected during the annual physicals conducted under anaesthesia using 13 mg/kg ketamine, delivered intramuscularly by the veterinarian team. Blood samples were stored at 4°C upon collection and shipped to Arizona State University where they were flash frozen into 0.5 mL aliquots and stored at −80°C.

### 2.2 Flow Cytometry

We followed our previously validated method for sorting primate cells from the faecal matrix using flow cytometry (Orkin et al. 2021), with a few modifications to optimize cell recovery. In brief, we homogenized the faecal samples in RNAlater by vortexing for 30 s, then centrifuged at 1727 rpm for 15 s to pellet the larger material. We transferred the supernatant to a 15 mL tube and filled it with Dulbecco's phosphate‐buffered saline (DPBS). We then filtered the supernatant through a 70 μm filter into a 50 mL tube. We transferred the resulting filtrate into a 15 mL tube and centrifuged at 1500 rpm for 5 min to pellet the cells. We washed the pellet twice with 13 mL of DPBS. Then, we resuspended the pellet in 300 μL of DPBS and filtered the solution again through a 35 μm filter into a 5 mL FACS tube. We prepared a negative control by mixing 250 μL of DPBS with 50 μL of the cell solution to account for autofluorescence. Next, we added 250 μL of 12 μM DAPI stain and 3 μL of AE1/AE3 Pan cytokeratin Alexa Fluor 488 antibody (ThermoFisher: 53‐9003‐82). We incubated the samples at room temperature for 15 min, followed by an incubation at 4°C for 15 min to 1 h, depending on time to initiate flow cytometry.

The cells were isolated using a BD FACSAria Fusion (BD Biosciences) flow cytometer at the University of Calgary Flow Cytometry Core with BD FACSDiva Software. Background fluorescence and cellular integrity were assessed by processing the negative control sample before all other prepared faecal samples. For each sample, we first gated the target population based on forward‐ and side‐scatter characteristics to minimize the presence of bacteria and cellular debris. Second, additional secondary and tertiary gates were applied to eliminate cellular agglomerations. Finally, we selected cells with antibody or DNA fluorescence that exceeded background levels. In instances where staining was ineffective, sorting was performed using only the first three gates.

### 2.3 DNA Extraction and Quality Assessment

Post sorting, we extracted DNA from faecal cells using the Arcturus PicoPure DNA Extraction Kit (Thermo Fisher Scientific, Kit # 0103), according to the manufacturer's instructions. We extracted DNA directly from urine (*Sapajus* only) stored in RNAlater using the same kit and protocol. Following the extractions, a cleanup step was carried out using Sera‐Mag Speedbeads (Fisher Scientific, catalogue # 09‐981‐123) at a 1.5:1 ratio. We extracted DNA from blood samples (*Sapajus*) at Arizona State University using the Qiagen DNeasy Blood & Tissue kits (Qiagen #69581) following the manufacturer's protocols.

### 2.4 Twist Targeted Methylation Sequencing

We prepared 147 libraries (Table 1) for enzymatic methyl sequencing involving a sequence capture approach following a modified Twist Targeted Methylation Sequencing (TMS) protocol (Twist Bioscience) optimized for non‐human primates. Detailed descriptions of the protocols can be found in Longtin et al. (2025). In brief, we used 200 ng of DNA as the input for library prep with NEBNext Enzymatic Methyl‐seq kit (P/N: E7120L). Library prep was modified to eight cycles of PCR for the final library amplification followed by a 0.65X SPRI bead cleanup. Libraries were then combined in equimolar amounts into pools of 12 (total concentration of 2000 ng per pool) for capture using the Human Methylome panel from Twist Biosciences following the manufacturer's instructions (P/N: 105521). Post‐hybridization libraries were then sequenced on the NovaSeq 6000 at the Vanderbilt Technologies for Advanced Genomics (VANTAGE) Core using 150 bp paired‐end sequencing, with a target of 30–50 M paired‐end reads per sample.

**TABLE 1:** Final sample size and demographic characteristics of the cohorts analysed in this study.

Species	Sample source	No. samples	No. individuals	Sex (F/M)	Age (mean ± SD)	Age range	Environment
*Cebus imitator*	Faecal	44 (age known)	43	30/13	12.19 ± 6.97	0.54–26.61	Wild
	Faecal	11 (age estimated)	10	3/7	~18 ± 6	~12–28	
*Sapajus apella*	Faecal	16	16	8/8	21.00 ± 7.96	9.90–42.84	Captive
	Blood	27	27	19/8	20.30 ± 7.12	9.96–41.64	
	Urine	18	17	15/2	20.00 ± 6.03	10.29–35.82	

*Note:* For wild *Cebus*, dates of birth were known to within a few days to 1 month for 81% (*n* = 43 monkeys). The remaining monkeys' ages were estimated based on morphological characteristics from the first time they were seen.

### 2.5 Sequence Alignment and Processing

Paired‐end FASTQ files were trimmed using Trim Galore! (function trim_galore with the flag ‐‐paired) https://www.bioinformatics.babraham.ac.uk/projects/trim_galore/ and mapped in bismark (Krueger and Andrews 2011) (functions bismark, ‐‐score_min L,0,‐0.6 ‐R 10\‐p 4) to the reference genome for the Panamanian White‐faced Capuchin Cebus_imitator‐1.0 (https://www.ncbi.nlm.nih.gov/datasets/genome/GCF_001604975.1/). We chose to map both species to the same reference genome because it was not possible to convert genomic coordinates from one species to the other due to the lack of chromosome‐level assemblies. Without this conversion, we would not be able to analyse methylation profiles across species and compare different types of samples, which is one of the aims of this study. Similar approaches have been used in other cross‐species DNA methylation (DNAm) comparisons (Vilgalys et al. 2019). Cytosine methylated and total counts were extracted with functions bismark_methylation_extractor and coverage2cytosine (flag ‐‐merge_CpG). Data quality assessments were performed using MultiQC (v1.7; Illumina). We assembled count data using the package *bsseq* (function read.bismark) (Hansen et al. 2012) for further processing and analysis in RStudio version 4.4.0 (RStudio Team 2022). We first removed samples with a conversation rate > 2% at CHH or CHG (*n* = 1) (indicative of poor conversion rate of unmethylated cytosines to thymine), then those with rate of mapping to the reference genome < 50% were excluded from further analysis (*n* = 19 samples: 6 faecal *Cebus*, 6 faecal *Sapajus*, and 8 urine) as well as one library with excessively high sequencing depth. Finally, we removed duplicated libraries (*n* = 10 excluded). The final sample size was 71 faecal, 27 blood, and 18 urine samples (Table 1).

The rate of mapping tended to be lower for *Sapajus* samples relative to *Cebus* samples, which was expected as our reference genome was the *Cebus* genome assembly (Wilcoxon rank sum test *W* = 2035, *p*‐value = 0.048, Figure S1). To ensure that any differences in the methylomes between species or sample sources would not result from this bias, we filtered CpGs in the four datasets separately (*Cebus*‐faecal, *Sapajus*‐faecal, *Sapajus*‐urine, and *Sapajus*‐blood) using a threshold of 5× in ≥ 75% of the samples in the set. This ensured that all CpGs included in the final data had adequate coverage in every species‐sample‐source subset. Then, we intersected the subsets for common CpG sites according to the type of data included for each analysis. For example, analyses using blood, faeces, and urine used the intersection of the four independently filtered datasets, while analyses using only faeces used the intersection of the two faecal sample subsets.

### 2.6 Methylation Signatures of Tissue Specificity

To identify the likely tissue source of host cells recovered from faeces, we compared the methylation profiles to DNAm single‐cell reference profiles. We anticipated capuchin‐derived profiles would primarily match intestinal epithelial signatures. We extracted the top 1000 cell‐specific markers identified by the HumanMethylationAtlas (Loyfer et al. 2023) for all cell‐types. We focused on cell‐specific hypomethylated loci, as they comprise 97% of markers identified by Loyfer et al. (2023). A cell‐specific marker is defined as a genomic region (one or more CpG sites) that exhibits markedly lower methylation levels in a given cell‐type compared to all other cell types.

Because of the lack of chromosome‐level assembly for the capuchin genome, we could not lift over the precise CpG coordinates from humans to capuchins, and instead had to rely on the curated information on genes (gene transfer format or gtf) to map promoters to analogous genes. We used Cebus_imitator.Cebus_imitator‐1.0.113.gtf to extract the location of genes annotated in both the human atlas and the capuchin genome. We passed the bed file coordinates of promoters to the function getCoverage() in *bsseq* with type = ‘Cov’ and ‘M’ with what = ‘perRegionTotal’ to calculate the coverage and methylation counts over entire promoters. We calculated promoter mean percent methylation across all capuchin samples and calculated the average difference in mean percent methylation at promoters annotated as markers for intestinal epithelia in the HumanMethylationAtlas versus all other cell types. We used 10,000 permutations of which promoters were assigned as markers of epithelia to create a random distribution of difference in percent methylation between a set of markers and the background of all other cell types. The one‐tailed *p*‐value was calculated by comparing the proportion of randomized differences larger than the observed difference.

To further validate our approach, we repeated the procedure of selecting promoters and testing for differences in methylation between marker and non‐marker sites using blood samples. Here, we investigated the top markers associated with circulatory immune cells versus other cell types from the HumanMethylationAtlas.

### 2.7 Biological Variables Recovered From Multidimensional Analysis of Methylation Profiles

We investigated correlates of methylation profiles using dimensionality reduction with Principal Component Analysis (PCA). We performed PCA using the prcomp() function with the scale argument set to FALSE on a matrix of percent methylation (*n* = 116 samples) using CpG sites after imputation of missing values using missMDA (Josse and Husson 2016) (*n* = 181,270 CpGs). Imputed values below and above the natural bounds were replaced with 0 (0.3% of values) and 1 (0.08% of values), respectively. To assess the relative explanatory power of the biological and technical covariates, we built linear regression models for PC1 and PC2. We included the four biological predictors (species, sex, age, and sample source) and three technical predictors in order to account for possible batch effects (batch, mapping efficiency, and percent methylation at CHG). The highest VIF was 2.6 in the full model suggesting limited collinearity (Zuur et al. 2010). We note that species and sample source, and batch and sample source remain partly confounded by design (Table S2), suggesting that fully disentangling the relative influence of these three parameters remains challenging. The mixed effect model including individual identity to account for the fact that a minority of individuals contributed several samples led to singular fit with a null variance explained by individual identity. An ANOVA showed no statistically significant difference (*p*‐value > 0.9) between a linear mixed and simple linear regression models. Therefore, we fitted models with lm() with z‐transformed covariates. Models' residuals were normally distributed as assessed using histograms and QQplots, and no influential data points were identified (DFbetas < 1 and DFFITs < 2). Pairwise significance was calculated with emmeans with Tukey correction for multiple testing (Lenth 2020) for categorical variables with more than two levels.

### 2.8 Multinomial Classifier of Sample Source

To examine further the discriminatory power of sample source (blood, faeces, urine) on methylation profiles, we used a multinomial regression algorithm in *glmnet* (Friedman et al. 2010; Tay et al. 2023) with a leave‐one‐out validation. The model was trained using cv.glmnet() on all samples but one by mapping sample source against methylation profiles at a set of 106,099 CpG sites with coverage across the four species‐sample‐source subsets after imputation with missMDA (Josse and Husson 2016). The penalization parameter lambda was internally determined by 10‐fold cross‐validation, and we used predict (type = ‘response’) with lambda.min on the test sample. The test sample is assigned probabilities that it originates from one sample source or the other. As covariates are not included in *glmnet*, this effectively tests the ability to determine sample source from DNAm profiles in the presence of unmodelled variation associated with sex, age, or technical batch effects.

### 2.9 Age Clocks Built From Faecal DNA Methylation Profiles

We used an elastic net regression in *glmnet* with a leave‐one‐out‐validation (LOOV) procedure to achieve the least biassed possible estimation of chronological ages based on methylation profiles following common practice in the field (Krieger et al. 2023; Lu et al. 2023). A first set of models were fitted on all samples (four species‐sample‐source combinations, *n* = 105) to assess model performance on a set of heterogeneous sample sources while maximizing sample size. A second set of models were fitted on faecal samples from the wild *Cebus* of known age (*n* = 44) to evaluate performance on a smaller sample size of homogeneous samples that speak to our goal of developing non‐invasive epigenetic clocks. For both scenarios, we started by imputing missing values using *missMDA* (Josse and Husson 2016) (function imputePCA with scale = TRUE and ncp = 2 as determined by estim_ncpPCA with scale = TRUE, ncp.min = 0, ncp.max = 5, method.cv = ‘Kfold’). Imputation was done independently in each subset, after excluding low variance sites constitutively hypo (average < 0.1) or hypermethylated (average > 0.9).

We next evaluated four modifications to the data preparation pipeline to optimize epigenetic clock accuracy, each motivated by biological or methodological considerations. One key modification addressed the known non‐linear relationship between DNA methylation and chronological age, particularly during early life stages when epigenetic changes occur more rapidly (Dabrowski et al. 2024; Horvath 2013; Lu et al. 2023). To account for this, we applied an age transformation that rescales ages relative to sexual maturity (set at 5 years) and compresses variation prior to this threshold. We compared models trained on untransformed ages (‘Classic’) to those using the transformed age scale (‘AgeTransfo’; Table S7). Second, we evaluated whether normalizing methylation values using a Yeo–Johnson transformation (bestNormalize package (Peterson 2021; Peterson and Cavanaugh 2020)) improved accuracy by reducing species or batch effects that may distort absolute methylation levels. We tested this both independently (Norm) and in combination with age transformation (AgeTransfoNorm). Third, we assessed whether restricting the feature space to CpG sites associated with age improved performance. Specifically, we selected sites with a Spearman correlation > 0.2 with age, as this approach has been shown to enhance clock accuracy (Dabrowski et al. 2024). These models are referred to as Corr (correlation filtering only) and CorrAgeTransfoNorm (correlation filtering combined with both age transformation and normalization). Finally, we tested whether excluding CpG sites showing species‐ or sample source‐specific differences—identified using binomial mixed models—improved cross‐species generalizability. This model, which combines bias filtering with age transformation and normalization, is referred to as NoBiasAgeTransfoNorm.

For all LOOV runs, the best penalization parameter lambda was determined internally using 10‐fold cross validation, and we ran iterations across values of alpha between 0 and 1 (i.e., spanning the space from ridge to lasso regression) and chose the alpha minimizing mean squared error (MSE). Model performance is reported based on Pearson's correlation coefficient and Median Average Error (MAE) expressed in years. For 24 individuals repeatedly sampled (one female *Cebus*, 15 female and eight male *Sapajus*) giving a total of 57 samples (22 blood, 18 faecal, 17 urine), we calculated the level of consistency across predicted ages by calculating for each individual the standard deviation of the absolute error between chronological age and predicted age. We report the mean and standard deviation of this within‐individual standard deviation across the 24 individuals.

### 2.10 Differential Methylation Between Species, Sample Source, and Sex

We tested for loci differential methylation using MACAU implemented in *PQLseq* (Lea et al. 2015, 2017; Sun et al. 2021). Our aim was to identify and remove loci (*N* = 69,353) which may lower clock's performance due to confounding effects of species, sample source, or sex on methylation levels. We ran binomial mixed models on data generated from faecal samples to test for a difference between *Cebus* and *Sapajus*, while accounting for dummy‐coded sex and age (models converged for *N* = 42,939 CpGs). Among samples collected from *Sapajus*, we tested for a difference in blood versus faecal and urine, while accounting for dummy‐coded sex and age (models converged for *N* = 58,697 CpGs). Finally, we tested for an effect of sex among faecal samples collected in *Cebus*, while accounting for age (models converged for *N* = 52,623 CpGs). All models included an identity matrix as a relatedness matrix, with relatedness set to 1 for samples from the same individual. For each set of results and after inspecting the distribution of *p*‐values, we applied False Discovery Rate (FDR) correction to account for multiple testing, using the *q* value approach developed by Storey et al. (2024). FDR corrections adjust significance thresholds to account for the expected proportion of false positives among significant results based on the number of tests run. From all three procedures, we extracted 20,313 sites which exhibited FDR > 0.05 in all cases (i.e., not statistically influenced by sex, sample source, or species) for the NoBiasAgeTransfoNorm elastic net regression model.

### 2.11 Age‐Associated Differences in Faecal Methylation Profiles

To test for loci differential methylation with age, we followed a similar procedure to the one described for species, sample source, and sex. Models estimating the effect of age on faecal methylation profiles included dummy‐coded sex, species, and batch as covariates, and were fitted using the betabinomial distribution in glmmTMB (Brooks et al. 2017). Models that did not converge (0.018%) were excluded from downstream analysis, leaving 183,274 loci (Table S13). We applied the same FDR correction by calculating *q*‐values from *p*‐values (Storey et al. 2024). To identify putative age‐associated differences in gene expression, we more closely examined a subset of 16,483 sites which overlapped with promoters from the capuchin gtf annotation (Table S14). The genomic location for the promoters were extracted from the capuchin's gtf file using makeTxDbFromGFF() and genes() in the package *GenomicFeatures*, and promoters (upstream = 2000, downstream = 200) from *IRanges* (Lawrence et al. 2013). We intersected these genomic coordinates with results from our analysis on age‐associated effect in capuchin (function findOverlap() with default settings). To allow comparisons with previous panmammalian studies, we also extracted the meta‐analysis effect sizes for age at CpG overlapping gene promoters from Lu et al. (2023) and annotated our results at overlapping promoters.

### 2.12 Pathway Enrichment

We tested for enrichment of molecular and cellular pathways among CpGs overlapping promoters. The effect sizes were averaged across CpGs overlapping the same promoters. We performed pathway enrichment analyses using fgsea() from the eponym package (Korotkevich et al. 2021; Subramanian et al. 2005) (arguments minSize = 15, maxSize = 500, nPermSimple = 10,000, eps = 0.0), with the subcollections Gene Ontology Biological Processes, Cellular Components, and Molecular Functions retrieved from msigdbr (species = ‘ *Homo sapiens* ’) using 1675, 1446, and 1543 genes ranked by effect sizes, respectively (Liberzon et al. 2011, 2015). For plotting, we simplified the results using collapsePathways() on pathways reaching an FDR < 0.05 and showed the top 20 pathways ranked by absolute normalized enrichment scores.

### 2.13 Transcription Factor Motif Enrichment

To be able to test for the presence of DNA motifs known to be associated with the binding of transcription factors, we first grouped single CpGs into differentially methylated regions (DMRs) using the pipeline from (Lea et al. 2016; Watowich et al. 2024) available at (https://github.com/mwatowich/Immune‐gene‐regulation‐is‐associated‐with‐age‐and‐environmental‐adversity‐in‐a‐nonhuman‐primate). DMRs were defined as segments including a higher‐than‐expected density of age‐associated sites exhibiting consistent direction of variation. First, we determined the number of CpG sites associated with age at a relaxed FDR threshold (FDR < 0.1) within 1000 base pairs of an age‐associated site (FDR < 0.05) at chance level by randomly permuting *p*‐values among CpGs (median of three loosely age‐associated CpGs per DMR). Therefore, we kept from the real data DMRs with at least four loosely age‐associated sites. Then, DMRs were removed if fewer than 80% of the CpGs in the DMR or CpGs at the loose FDR threshold were in the same direction. We filtered 18 DMRs which were longer than 99% of the distribution, leaving 1762 DMRs, which were 485 ± 459 base pairs long on average (range = 9–2468), and included 26 ± 25 CpGs (range = 4–185), of which 16 ± 17 and 13 ± 15 were loosely and strictly age‐associated respectively. We defined a background set of DMRs by applying no threshold on significance and concordance of age‐associated variations, and further removing the set of previously identified DMRs from the background set. Finally, we filtered background regions longer than the longest DMR (*n* = 87 regions removed) to obtain more homogeneous sets (8470 background regions of average length 441.6 ± 444.8, range = 7–2467).

We tested for transcription factors binding site motifs (TFBSs) enrichments using *monaLisa* (Machlab et al. 2022). We downloaded vertebrates' transcription factor binding site position weight matrices from Jaspar 2020 (Fornes et al. 2020). Region sequences were extracted from the capuchin genome converted to a FAFile using getSeq() from *BSgenome* (Pagès 2025). We compared hypermethylated DMRs (*n* = 1630) and hypomethylated DMRs (*n* = 132) to the background set. TFBSs with adjusted *p*‐value < 0.001 are shown.

### 2.14 Overlap With Age‐Associated Sites in Pan Mammalian EWAS of Age

We extracted the top 1000 CpGs exhibiting higher and 1000 CpGs exhibiting lower methylation with age in an EWAS across several tissues and species of eutherian mammals from Lu et al. (2023). We reasoned that these top age‐associated sites are more likely to be shared broadly. Because we could not map CpGs from the pan mammalian array to the capuchin genome directly, we relied on gene annotations. Specifically, we used gene names from the pan mammalian data to extract the genomic location for the promoters and exons present in the capuchin's gtf file (package *IRanges* functions promoter() and exonsBy() with by = gene). We intersected these genomic coordinates with results from our analysis on age‐associated variations in capuchin (function findOverlap() with default settings). We did not apply any filtering for significance as we are interested in the consistency of the direction of difference broadly. Also, note that some genes could be associated with several CpGs, which may or may not differ in the same direction with age. To compare the direction of age effect in the capuchin and pan mammalian dataset, we had to ensure that each gene would be represented by a unique direction of effect with age in the pan mammalian data. Genes that were unambiguously associated with effect sizes all in one direction were first selected (*n* = 116). Then, for genes associated with several CpGs with effects in opposite directions (*n* = 237), we calculated the proportion of sites that are higher with age. After visual inspection of the distribution, we decided to keep sites exhibiting a proportion of positive differences < 25% and > 75% (*n* = 113). Sites falling between these boundaries were excluded. We assigned the direction of effect of the majority of CpGs overlapping the gene. We performed a sensitivity check by running the analyses using 0–1, 10–90, and 40–60 as alternative cutoffs. This procedure allowed us to assign either a positive or negative direction of age effect for 816 genes out of the 851 originally present in the pan mammalian dataset. We then intersected the datasets to compare the direction of age effect for 337 genes common to both datasets (*n* = 9731 CpGs, with an average 28.9 ± 33.4 CpGs overlapping a gene in capuchins) (Table S19).

## 3 Results

### 3.1 Non‐Invasive Sample‐Derived Methylation Signatures Reflect Tissue Specificity

We efficiently captured DNA methylation (DNAm) profiles from faecal samples by innovating a protocol uniting Fluorescence‐Activated Cell Sorting (fecalFACS) (Orkin et al. 2021) plus Twist Targeted Methylation Sequencing (TTMS)—a capture based approach that includes enzymatic methyl sequencing. TTMS uses probes designed to cover ~4 million CpG sites in the human genome, and we recently demonstrated that this human‐based commercial probe set could capture ~2 million CpG sites in high‐quality non‐human primate samples (Longtin et al. 2025) (Table S1). With this novel, combined approach, we covered 905,950 sites in faecal samples from wild‐living *Cebus*, and 1,245,571 sites in faecal samples from captive‐living *Sapajus* at a coverage of 5× in ≥ 75% of the samples in the set. This represents about half of the sites recovered from blood samples in this species (Longtin et al. 2025), likely due to the more fragmented nature of DNA extracted from faecal samples. After filtering (Table 1), we had high coverage of ~1 million CpG sites: mean coverage ± SD was 72.21X ± 87.70 in *Sapajus* blood, 184.41X ± 255.53 in *Sapajus* faecal, 161.25X ± 219.63 in *Cebus* faecal, and 316.94X ± 521.37 in *Sapajus* urine samples. For joint analyses across all sample types, we focus on 711,737 shared CpGs with similar coverage across four datasets.

DNAm patterns are highly cell‐type specific, meaning that methylation profiles measured from bulk tissues or blood primarily reflect the underlying composition of constituent cell populations. This property is well established in conventional biological samples but becomes more challenging to interpret in unconventional sample types such as faeces and urine, where the diversity and relative abundance of contributing cell types are less well characterized. Having obtained DNAm data spanning up to 1 million CpG sites (Table 1), we next sought to verify whether the resulting profiles matched the expected biological sources. We anticipated that host cells recovered from faeces would predominantly originate from the intestinal epithelium.

**FIGURE 2:**
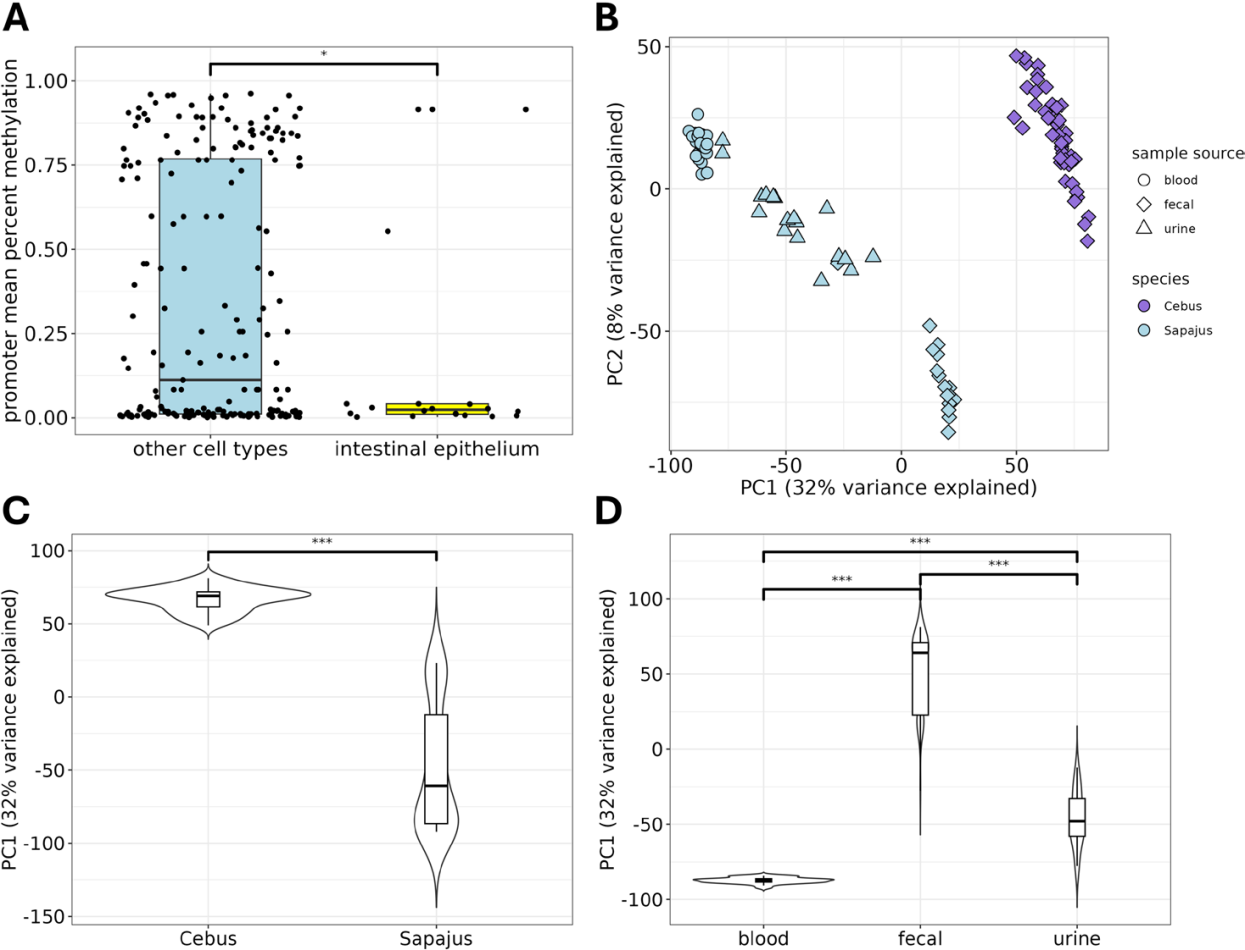
Faecal‐derived DNAm profiles capture meaningful biological variation from tissue and species of origin. (A) DNAm profiles measured from non‐invasively collected faecal samples most closely match the DNAm hypomethylation patterns of intestinal epithelial cells. (B) Projection of methylation profiles along the first components of a PCA. Species (C) and sample source (D) clustered along the first principal components. *p*‐values are reported from a linear regression including biological and technical covariates, followed by post hoc pairwise comparisons with correction for multiple testing for factors with more than two levels. In boxplots, boxes represent the interquartile range (IQ), which contains the middle 50% of the records, and a line across the box indicates the median. Vertical lines extend from the upper and lower edges of the box to the highest and lowest values which are no greater than 1.5 times the IQ range. Violin plots display the data distributions and full ranges. *p*‐values are coded as *< 0.05, **< 0.01, and ***< 0.001.

To test the tissue specificity, we compared our datasets to a multi‐tissue DNAm reference atlas (see Methods), focusing on promoter regions exhibiting cell type‐specific hypomethylation (Table S3). For each sample type, we expected the corresponding tissue of origin (e.g., blood cells in blood‐derived samples) to show the strongest hypomethylation signal at its matched marker genes. Consistent with this expectation, blood samples showed low methylation at promoters of genes identified as blood cell markers (314 gene promoters in capuchins mapped to 439 human cell‐type markers; 10,000 random permutations, one‐tailed *p* = 0.03; *n* = 2,351,140 CpGs; Figure S2). Using faecal DNAm profiles (*n* = 751,398 CpGs), we mapped 165 capuchin gene promoters to 231 human promoters associated with specific tissues or cell types (i.e., on average, each capuchin promoter corresponded to 1.32 ± 0.63 cell types). These faecal‐derived methylation profiles also matched expectations, showing the lowest methylation at marker loci for intestinal epithelial cells (Figure 2A; 10,000 random permutations, one‐tailed *p* = 0.03). We were unable to assess the tissue of origin for urinary samples due to the absence of a urinary tract reference atlas (Supplemental Results and Figure S2). Together, these results demonstrate that (1) DNAm can be reliably quantified from non‐invasively collected samples, and (2) the resulting methylation profiles reflect the biological signatures of the originating host cells in situ.

### 3.2 DNAm Profiles From Multiple Tissue Sources Reflect Species, Sex, and Age

We tested whether DNAm profiles would carry meaningful biological signatures, namely of species, age, sex, and sample types. To that end, we used dimensionality reduction by PCA on all samples from individuals for which date of birth was known to within 1 month, thereby excluding 11 samples from 10 *Cebus* (Table 1; *n* = 105 samples, 181,270 CpGs). The first two PCA components (cumulative variance explained = 40%) clearly separated the two species and sample sources (Figure 2B–D, Figure S3, and Tables S4–S5). Age and sex were also associated with the first two components (Figure S4). Of the technical predictors, only the batch exhibited some degree of association with PC2 (Figure S5; Tables S4–S5). Clustering by sample source was also evident when restricting the PCA to *Sapajus* samples to reduce the confound between species, sample source, and batch (Figure S6). Using a multinomial classifier, we could also identify the source of blood, faecal, and urine samples with 96% accuracy (Supplemental Results and Table S6).

### 3.3 Accurate Age Prediction From Faecal‐Derived DNAm Profiles

**FIGURE 3:**
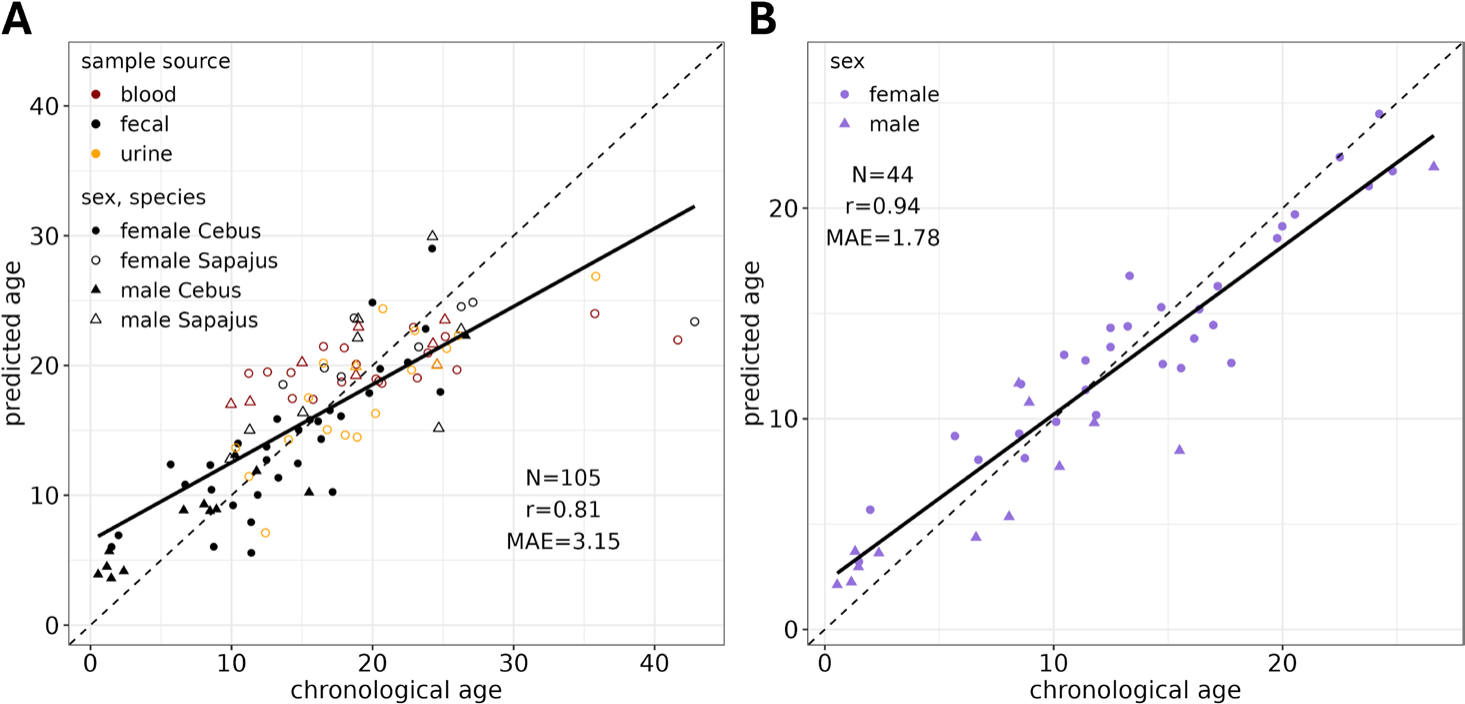
Accurate age estimation from faecal‐derived methylation profiles. (A) DNAm clock trained on blood, faecal, and urine samples in two capuchin species. Age predictions are from a leave‐one‐out validation. (B) Faecal DNAm clock in *Cebus imitator* estimates the age of wild‐living individuals from non‐invasively collected samples. Model performance is indicated by Pearson's correlation coefficient (*r*) and Median Average Error (MAE). The solid lines are the best‐fit linear regression of predicted ages on chronological ages, while the dotted lines reflect the line of identity (*x* = *y*).

Given the individual‐representative biological signatures in the DNAm profiles, we built two DNAm clocks to predict the age of samples based on methylation profiles using elastic net regression. The first clock was trained on all samples from both species (*n* = 105 samples; 69,353 CpGs), maximizing sample size and incorporating data from blood, faecal, and urine samples. The clock (model ‘AgeTransfoNorm’, cf. Table S7) was strongly predictive of age, estimating age to within a median of 3.15 years (Pearson's *r* = 0.81 between chronological and predicted age; Figure 3A; Tables S8, S9). Predictions also showed strong reliability within individuals, with a mean ± SD of 1.69 ± 0.83 years discrepancy across predicted ages for 24 individuals for which we had samples from multiple biological sources. Prediction accuracy was robust to alternative preprocessing steps—such as excluding CpGs with species, sample source, or sex‐associated methylation differences (Methods; Supplemental Results and Figures S7A and S8).

Currently, one of the most pressing challenges for the field is to develop entirely non‐invasive techniques for age estimation. To address this challenge, we built a second clock only from faecal samples from the free‐ranging white‐faced capuchins ( *Cebus imitator* ) (*n* = 55 samples; 229,020 CpGs). Despite being developed with a smaller sample size, our *Cebus* faecal clock (model ‘AgeTransfo’, cf. Table S7) had even higher accuracy than our all‐sample clock: with a correlation of 0.94 and median absolute error (MAE) 1.78 years (Figure 3B; Figures S7B and S8, Tables S10–S11 and Supplemental Results). When applied to the 11 samples from monkeys with morphologically estimated ages, the clock was less accurate than in monkeys of known age (MAE = 3.5 years; Supplemental Results and Table S12), likely due to imprecision in the morphological estimates.

### 3.4 Faecal‐Derived Methylation Profiles Captures Age‐Associated Differences

**FIGURE 4:**
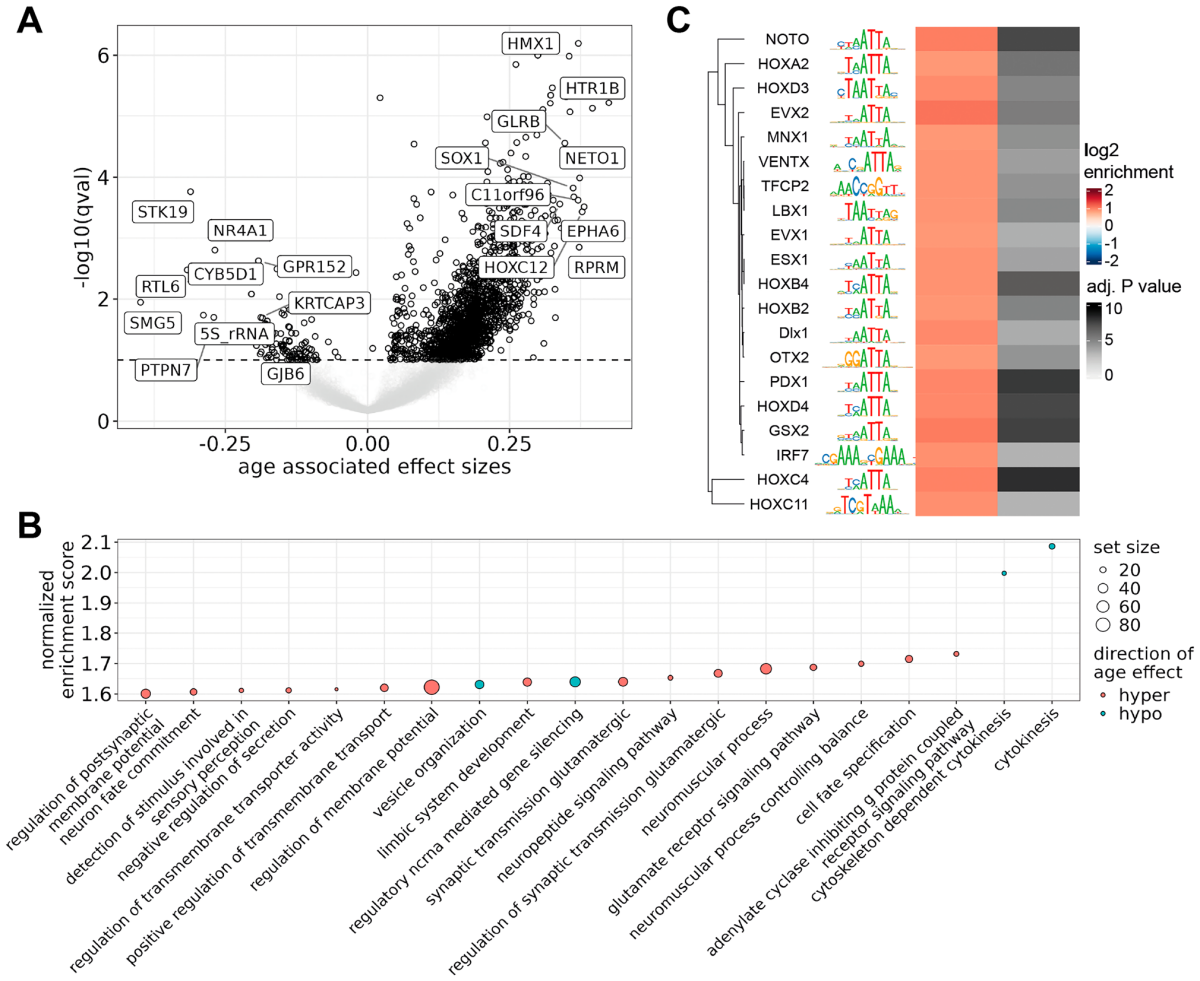
Age is associated with differences in the methylome. (A) Age‐associated differences in methylation levels measured from faecal samples. CpG sites overlapping with promoters in the capuchin genome are represented on the forest plot, with sites reaching FDR < 0.1 in black. Top age‐associated sites are labelled for illustrative purposes, and include several homeobox genes and protein‐coding genes involved in neoplasic processes. (B) GO enrichment analysis of the genes overlapping age‐associated sites are shown. The 20 most enriched GO terms are shown on the rows, the dot sizes show the number of enriched genes, and the *x*‐axis shows the proportion of enriched genes relative to all genes associated with the GO term (absolute normalized enrichment score). All enrichments have Bonferroni‐adjusted *p*‐value < 0.05. (C) Top transcription factor binding site enrichment among regions exhibiting higher methylation levels at older ages. The 20 most strongly enriched motifs with adjusted *p*‐value < 0.001 are shown.

We tested for age‐associated DNAm differences at 183,274 CpG sites across 60 faecal samples (combined across species). Age was associated with DNAm at 15.5% of tested sites (*n* = 28,372, FDR < 0.05; Table S13). To focus on regions with strong cis‐regulatory potential, we mapped 16,483 of these sites to gene promoters (Figure 4A; Table S14). These age‐associated promoters were associated with genes involved in transcriptional regulation, intracellular signalling, immune function, and neural development. For instance, 49 of 57 CpGs in the RPRM promoter—part of the p53 pathway and implicated in gastric cancer risk via methylation‐mediated silencing—were hypermethylated (Liu and Yang 2015; Wang et al. 2016; Wu and Du 2022). Gene ontology analysis further supported these patterns, showing that age‐associated hypermethylation was enriched in pathways related to neural function, membrane excitability, development, and cell differentiation (Figure 4B, Figure S9; Tables S15–S17), consistent with previous reports (Lu et al. 2023). In contrast, hypomethylated sites included regulation of proliferative cell cycle (De Cecco et al. 2019; Page et al. 2022) (Figure 4B, Figure S9; Table S15). There was no strong evidence for coordinated methylation variation in either direction among immune pathways.

To investigate potential regulatory impacts, we grouped CpGs into regions and assessed enrichment for transcription factor binding sites (TFBSs) in regions that become hypermethylated with age. We identified 146 enriched and one under‐enriched TFBS, including several involved in development (ZBTB14, HES1, HES2), cell cycle control (TFDP1), and metabolism (NRF1, ARNT::HIF1A) (Figure 4C and Table S18). When comparing these results to the 38 TFBSs previously found to be hypermethylated with age in rhesus macaques (Watowich et al. 2024), we observed that 31 were also hypermethylated in capuchins, suggesting some degree of conservation across primates. No enriched TFBSs were detected in hypomethylated regions in our dataset.

As an external validation and to assess cross‐species conservation, we compared our age‐associated CpGs to those identified in a recent multi‐tissue panmammalian epigenome‐wide association study (EWAS) (Lu et al. 2023). Of 9731 CpGs across 337 genes that could be compared, 70.9% of CpGs that had higher methylation with age in capuchins showed similar directional age‐associated effect in the panmammalian EWAS, and 73.8% of CpGs that had lower methylation were similarly consistent (Fischer's Exact Test for consistency in the direction of age effect: OR = 5.7, 95% Confidence Interval = 5.1–6.3, *p*‐value < 0.001; Sensitivity check: 5.7 < OR < 6.0, all *p*‐values < 0.001; Table S19).

## 4 Discussion

Accurate information on chronological and biological age is essential across animal research, from behavioural ecology to conservation and biogerontology. In this study, we generated DNA methylation (DNAm) profiles from cells sorted from non‐invasively collected faecal samples in a population of capuchin monkeys that have been studied continuously for over 40 years in their natural environment (Melin et al. 2020). We show that faecal DNAm profiles derived from intestinal epithelial cells retain strong biological signal: they differentiate among tissue types, species, and sexes, enable precise estimation of chronological age, and capture age‐associated epigenomic variation in a wild non‐human primate.

### 4.1 High‐Quality Methylation Profiles Are Recovered From Faecal Samples

Faecal DNAm profiles exhibited canonical hypomethylation patterns characteristic of epithelial cells of the lower gastrointestinal tract (Loyfer et al. 2023). Although faecal and urinary DNA likely derive from heterogeneous cell populations (Banila et al. 2023; Loyfer et al. 2023), methylation profiles appear sufficiently preserved to support biological inference. This creates opportunities to address both broad systemic questions and more targeted studies of gut physiology, host‐microbiome interactions, and metabolic processes (Anderson et al. 2024; Lea et al. 2016; Sadoughi et al. 2022; Watowich et al. 2024)—many of which are central to aging and disease.

We also identified clear species‐ and tissue‐specific DNAm signatures, distinguishing closely related capuchin species as well as faeces, blood, and urine samples. DNAm is known to reflect both phylogenetic distance and tissue identity (Blake et al. 2020; Haghani et al. 2023; Loyfer et al. 2023), and our results show that these signals are preserved in non‐invasively collected samples under field conditions. This supports the broader applicability of this approach across taxa, although standardizing protocols will be important to maximize reproducibility and sensitivity across studies.

### 4.2 Non‐Invasive and Accurate Estimation of Chronological Age Using Faecal Samples

Crucially, faecal samples can be used to estimate chronological age with high accuracy (median error: 1.59 years), representing a substantial improvement over previous faecal‐based methods in other species, including dolphins (Yagi et al. 2024) and mice (Hanski et al. 2024), as well as a microbiome‐based clock in baboons (Dasari et al. 2024). The accuracy achieved is comparable to DNAm clocks developed in wildlife from blood and other tissues (Lu et al. 2023). We also observed discrepancies between DNAm‐based and observer‐estimated ages, likely reflecting imprecision in morphology‐based estimates. This underscores the utility of non‐invasive epigenetic clocks for improving age estimation in populations where a subset of ages are unknown. More broadly, this approach opens new possibilities for non‐invasive research—from estimating the ages of unmonitored individuals to exploring how social, environmental, and physiological factors shape aging trajectories.

### 4.3 Age‐Associated Variation in the Epigenome of Non‐Human Primates in Their Natural Environment

Beyond age prediction, we identified thousands of CpG sites with methylation levels associated with age. Sites located in gene promoters were enriched for pathways related to development, neural function, membrane excitability, and cell differentiation. This pattern suggests that a substantial portion of the age‐associated variation captured across this broad age range reflects underlying developmental and regulatory processes. These findings are consistent with, and largely conserved relative to, recent panmammalian EWAS results (Lu et al. 2023), reinforcing the comparative relevance of field‐based studies. Together, these results support the view that aging involves conserved molecular signatures, including regulatory changes in developmental pathways. This interpretation aligns with evolutionary theories of aging, such as antagonistic pleiotropy, in which genes that are beneficial early in life can have deleterious effects later on (de Magalhães 2023; Lemaître et al. 2015). Understanding how these epigenetic patterns emerge during early life will be key to explaining variation in aging trajectories.

### 4.4 Epigenomic From the Lab to the Field

Growing evidence in humans shows that environmental factors shape morbidity and survival, potentially through their effect on the pace of aging (Argentieri et al. 2025; Kivimäki et al. 2025), yet the relative importance of specific exposures and individual susceptibility remains unclear. Long‐term studies of wild primates are well‐positioned to address this gap, having already linked social dynamics, early life adversity, hormone profiles, and ecological conditions to later‐life outcomes (Boesch et al. 2000; Campos et al. 2024; Emery Thompson et al. 2020; Melin et al. 2020; Newman et al. 2023; Perry et al. 2012; Wilson et al. 2020). Epigenetic data—particularly from blood—has begun to reveal how such exposures leave molecular traces (Anderson et al. 2024; Sadoughi et al. 2026; Watowich et al. 2024). However, although many long‐term studies obtain blood samples, doing so is difficult, risky, or prohibited for many species, especially if repeat sampling is desired. Non‐invasive sampling therefore provide a critical extension, enabling the study of epigenetic in species for which invasive sampling is not feasible, while also improving the safety and ease of longitudinal sampling in established long‐term studies (Vullioud et al. 2024; Weber et al. 2024; Yagi et al. 2024).

Within this context, capuchins are emerging as a promising non‐human primate model of aging (Campos et al. 2024). Their large brain size, propensity for innovation, and evidence of positive selection for longevity‐associated genes suggest that the mechanisms underlying their long lives may share commonalities with those in humans (Byrne et al. 2022; Orkin et al. 2021). Variation in survival driven by ecological and social factors, including climate (Carrera et al. 2025), creates opportunities to link environmental exposures to molecular measures of aging. Incorporating DNAm data with longitudinal survival records will be particularly valuable for testing whether epigenetic variation predicts lifespan and health across individuals in species other than humans (Froy et al. 2021; Lemaître et al. 2024; Vedder et al. 2022).

## 5 Conclusion

Although we have demonstrated the value of non‐invasive DNAm profiling, we acknowledge some limitations. As in other machine learning‐based clocks, age predictions showed mild bias, with overestimation in younger individuals and underestimation in older ones. This likely reflects regression to the mean as well as demographic processes such as selective disappearance (El Khoury et al. 2019; Lea et al. 2017). Estimated ages for older individuals should be regarded as a lower bound, and increasing sample size will help reduce this bias. Importantly, the clock is very accurate in predicting the ages of most mature individuals who may, for example, immigrate into long‐term study populations. Our sample size also limits power to detect associations among multiple variables, particularly given partial collinearity between sample source and batch effects. Nevertheless, the consistency of observed biological signals with prior work supports the robustness of our findings (Blake et al. 2020; Loyfer et al. 2023). While faecal DNAm performs well within and across closely related species, dietary and digestive differences may limit its comparability in other taxa. Notably, wild and captive capuchins remained comparable despite major differences in diet, indicating robustness within this system; however, validation in more other taxa, such as carnivores, is still needed to assess the taxonomic breadth of the applicability of this approach.

Overall, our results demonstrate that non‐invasively collected faecal samples provide a reliable source of DNAm profiles for studying aging in wild populations. Incorporating epigenetic tools into field research has the potential to bolster our understanding of aging across diverse ecological contexts and species. Expanding these approaches to underrepresented taxa with high translational relevance will be critical for understanding the evolution and variability of aging, as well as its broader relevance to health and disease. More broadly, non‐invasive epigenetic approaches can accelerate discovery across domains, from developmental plasticity and resilience to environmental stressors and the evolution of aging in natural contexts.

## Author Contributions

Conceptualization: Amanda D. Melin, Noah Snyder‐Mackler; Methodology: Amanda D. Melin, Noah Snyder‐Mackler, Raquel Hernández‐Rojas, Hadjira Hamou, Joseph D. Orkin, Baptiste Sadoughi; Validation: Amanda D. Melin, Noah Snyder‐Mackler, Sarah F. Brosnan, Baptiste Sadoughi; Formal analysis: Baptiste Sadoughi, Raquel Hernández‐Rojas, Hadjira Hamou; Investigation: Amanda D. Melin, Baptiste Sadoughi, Noah Snyder‐Mackler, Raquel Hernández‐Rojas, Hadjira Hamou; Resources: Amanda D. Melin, Katharine M. Jack, Fernando A. Campos, Sarah F. Brosnan, James P. Higham, Noah Snyder‐Mackler; Data collection: Ronald Lopez, Sierra M.V. Simmons, Megan Mah, Elizabeth Slikas; Data curation: Fernando A. Campos, Amanda D. Melin, Raquel Hernández‐Rojas, Hadjira Hamou, Megan Mah, Noah Snyder‐Mackler, Baptiste Sadoughi; Writing – original draft: Baptiste Sadoughi, Amanda D. Melin, Noah Snyder‐Mackler; Writing – review and editing: all authors; Visualization: Baptiste Sadoughi, Amanda D. Melin; Supervision: Amanda D. Melin, Fernando A. Campos, Katharine M. Jack, Noah Snyder‐Mackler; Project administration: Amanda D. Melin, Fernando A. Campos, Katharine M. Jack, Noah Snyder‐Mackler, Megan Mah; Funding acquisition: Amanda D. Melin, Fernando A. Campos, Katharine M. Jack, James P. Higham, Joseph D. Orkin, Noah Snyder‐Mackler; All authors read and approved the manuscript.

## Funding

This work was supported by funding from the Natural Sciences and Engineering Research Council of Canada (NSERC RGPIN‐2017‐03782 to A.D.M., C.G.S. to M.E.M.), the Canada Research Chairs program (950‐231257 to A.D.M.), the National Institutes of Health Aging Research In Animals (NIH ARIA) program (R61‐AG078529‐01 to F.C., A.D.M., K.J., J.P.H., J.D.O., N.S.M.), and the National Science Foundation (NSF BCS 2127375 and NSF SES 1919305 to S.F.B.).

## Disclosure

Benefits Generated: This research built on and strengthened international collaborations among researchers in Canada, Costa Rica and the USA. It further promoted high‐quality training, methods development and skills acquisition in each country. Further benefits from this research accrue from the sharing of our data and results on public databases as described above. The research addresses a priority concern regarding the establishment of naturalistic models of aging and the refining of molecular tools for the non‐invasive study of wildlife. These methods will promote wildlife conservation through non‐invasive study and open new doors for geroscience research.

## Ethics Statement

All work adheres to the joint Principles for the Ethical Treatment of Non‐human Primates as set forth by the American Society of Primatologists and International Primatological Society. Faecal samples from wild capuchins in Costa Rica were collected under permits issued by the Animal Care Committee (ACC) of the University of Calgary in Canada (AC19‐0167/AC24‐0021), and by the Sistema Nacional de Áreas de Conservación (SINAC) and the Área de Conservación Guanacaste (ACG: R‐SINAC‐ACG‐PI‐059‐2022/ACG‐PI‐033‐2023ACG‐PI‐011‐2024), and CONAGEBIO (R‐013‐2022‐OT‐CONAGEBIO/R‐042‐2023‐OT‐CONAGEBIO) in Costa Rica. Faecal samples were imported to Canada under Canadian Food Inspection Agency (CFIA) permits A‐2023‐06194‐1 and A‐2022‐05488‐4. Blood, faecal and urine samples were collected from captive brown capuchins ( *Sapajus apella* ) at Georgia State University, under IACUC protocol (A20018). Faecal and urine samples were imported to the University of Calgary, Canada, under CFIA permit A‐2024‐03380‐4.

## Conflicts of Interest

The authors declare no conflicts of interest.

## Supporting information


**Figure S1:** Mapping efficiency in Cebus and Sapajus across batches. (A) Mapping was lower in Sapajus, which could be partially explained by the fact that the reference genome used for alignment was from Cebus. Boxes represent the interquartile range (IQ), which contains the middle 50% of the records, and a line across the box indicates the median. Vertical lines extend from the upper and lower edges of the box to the highest and lowest values which are no greater than 1.5 times the IQ range. (B) Batch effects coupled with uneven representation of each species across batches might also contribute to the apparent species differences.
**Figure S2:** Percent methylation at cell‐specific hypomethylated markers. (A) In blood and (B) in urine samples. Cell‐specific hypomethylated markers were defined based on the HumanMethylationAtlas. Boxes represent the interquartile range (IQ), which contains the middle 50% of the records, and a line across the box indicates the median. Vertical lines extend from the upper and lower edges of the box to the highest and lowest values which are no greater than 1.5 times the IQ range.
**Figure S3:** Projection of methylation profiles along the second components of a PCA. (A) Species and (B) sample source differentially cluster along PC2. In boxplots, boxes represent the interquartile range (IQ), which contains the middle 50% of the records, and a line across the box indicates the median. Vertical lines extend from the upper and lower edges of the box to the highest and lowest values which are no greater than 1.5 times the IQ range. Violin plots display the data distributions and full ranges. *p*‐values are coded as *< 0.05, **< 0.01, and ***< 0.001.
**Figure S4:** Projection of methylation profiles along the first two components of a PCA. (A) Projection of samples on PC1 and PC2. Samples differentially load according to sex on PC1 (B) and PC2 (C). Individual's age at sampling also correlates with loading on PC1 (D) and PC2 (E). In boxplots, boxes represent the interquartile range (IQ), which contains the middle 50% of the records, and a line across the box indicates the median. Vertical lines extend from the upper and lower edges of the box to the highest and lowest values which are no greater than 1.5 times the IQ range. Violin plots display the data distributions and full ranges. *p*‐values are coded as *< 0.05, **< 0.01, and ***< 0.001.
**Figure S5:** Batch effects on methylation profiles. (A) Projection of samples on PC1 and PC2. Batches loading are plotted against (B) PC1 and (C) PC2. *p*‐values were calculated using post hoc pairwise comparisons on adjusted marginal means in package *emmeans* with Tukey correction for multiple testing (*n* = 4). In boxplots, boxes represent the interquartile range (IQ), which contains the middle 50% of the records, and a line across the box indicates the median. Vertical lines extend from the upper and lower edges of the box to the highest and lowest values which are no greater than 1.5 times the IQ range. Violin plots display the data distributions and full ranges. *p*‐values are coded as *< 0.05, **< 0.01, and ***< 0.001.
**Figure S6:** Projection of methylation profiles from *Sapajus* samples along the first two components of a PCA.
**Figure S7:** Performance of methylation clocks. Elastic net regression algorithms were trained on (A) all sample types, and (B) *Cebus* faecal samples only with different levels of pre‐processing. Models are ordered on the *x*‐axis per increasing Median Average Error (MAE) expressed in years.
**Figure S8:** Improvements of the clock trained on all samples. The best performing version of the clock trained on all sample sources was rerun after excluding the oldest individuals in order to match age ranges between clock types. Model performance is indicated by Pearson's correlation coefficient (cor) and MAE, with N the sample size. The full lines show a perfect match between chronological and predicted ages, and the dotted lines the linear regression of predicted ages on chronological ages.
**Figure S9:** Pathways enrichment with age. Top age‐associated enriched pathways for (A) biological processes, (B) molecular functions, and (C) cellular components. The top 25 positively and 25 negatively enriched pathways are plotted.

**Table S1:** Sample metadata and library quality scores.
**Table S2:** Sample size for pairs of categorical variables in the dataset.
**Table S3:** List of CpG sites overlapping promoters of genes considered as tissue or cell‐specific markers in Loyfer et al. (2023).
**Table S4:** Linear correlates of multidimensional PC1 and PC2 loadings.
**Table S5:** Posthoc pairwise comparisons for categorical variables.
**Table S6:** Classification of sample source using machine learning multinomial models.
**Table S7:** glmnet model preprocessing pipeline.
**Table S8:** Coefficients of the clock trained on blood, faecal, and urine samples.
**Table S9:** Age predictions from the clock trained on blood, faecal, and urine samples.
**Table S10:** Faecal clock coefficients.
**Table S11:** Age predictions from the faecal clock.
**Table S12:** Age predictions from the faecal clock for individuals with uncertain ages.
**Table S13:** EWAS for the effect of age on faecal sample methylation profiles.
**Table S14:** Annotation of age‐associated CpG sites overlapping promoters.
**Table S15:** Gene Ontology Biological Processes enrichment analysis.
**Table S16:** Gene Ontology Cellular Components enrichment analysis.
**Table S17:** Gene Ontology Molecular Functions enrichment analysis.
**Table S18:** Enrichment for transcription factor binding motifs among age‐associated hypermethylated differentially methylated regions.
**Table S19:** Comparison of age EWAS in faecal samples from capuchins with age EWAS from a panmammalian multi‐tissue analysis in Lu et al. (2023) at a subset of common genes.

## Data Availability

Genomic sequences and associated metadata generated as part of this study have been deposited in NCBI's Sequence Read Archive under accession number PRJNA1272643. Data previously deposited as part of another project can be found under accession number PRJNA1156067. R code and bash command lines are available from github https://github.com/BaptisteSadoughi/CapuchinsDNAm.
